# Laparoscopic versus hybrid approach for treatment of incisional ventral hernia: a prospective randomised multicentre study, 1-year results

**DOI:** 10.1007/s00464-019-06735-9

**Published:** 2019-04-02

**Authors:** Mirella Ahonen-Siirtola, Terhi Nevala, Jaana Vironen, Jyrki Kössi, Tarja Pinta, Susanna Niemeläinen, Ulla Keränen, Jaana Ward, Pälvi Vento, Jukka Karvonen, Pasi Ohtonen, Jyrki Mäkelä, Tero Rautio

**Affiliations:** 1 Department of Surgery, Oulu University Hospital, Oulu, Finland; 2 Department of Radiology, Oulu University Hospital, Oulu, Finland; 3 Department of Surgery, Helsinki University Hospital, Helsinki, Finland; 4 Department of Surgery, Päijät-Häme Central Hospital, Lahti, Finland; 5 Department of Surgery, Seinäjoki Central Hospital, Seinäjoki, Finland; 6 Department of Surgery, Valkeakoski Regional Hospital, Valkeakoski, Finland; 7 Department of Surgery, Kymenlaakso Central Hospital, Kotka, Finland; 8 Department of Surgery, Turku University Hospital, Turku, Finland; 9 Division of Gastroenterology, Department of Surgery, Oulu University Hospital, OYS, PL 21, 90029 Oulu, Finland

**Keywords:** Incisional ventral hernia, Laparoscopy, Hybrid, Recurrence, Quality of life, Chronic pain

## Abstract

**Purpose:**

Laparoscopic incisional ventral hernia repair (LIVHR) is often followed by seroma formation, bulging and failure to restore abdominal wall function. These outcomes are risk factors for hernia recurrence, chronic pain and poor quality of life (QoL). We aimed to evaluate whether LIVHR combined with defect closure (hybrid) follows as a diminished seroma formation and thereby has a lower rate of hernia recurrence and chronic pain compared to standard LIVHR.

**Methods:**

This study is a multicentre randomised controlled clinical trial. From November 2012 to May 2015, 193 patients undergoing LIVHR for primary incisional hernia with fascial defect size from 2 to 7 cm were recruited in 11 Finnish hospitals. Patients were randomised to either a laparoscopic (LG) or a hybrid (HG) repair group. The main outcome measure was hernia recurrence, evaluated clinically and radiologically at a 1-year follow-up visit. At the same time, chronic pain scores and QoL were also measured.

**Results:**

At the 1-year-control visit, we found no difference in hernia recurrence between the study groups. Altogether, 11 recurrent hernias were found in ultrasound examination, producing a recurrence rate of 6.4%. Of these recurrences, 6 (6.7%) were in the LG group and 5 (6.1%) were in the HG group (*p* > 0.90). The visual analogue scores for pain were low in both groups; the mean visual analogue scale (VAS) was 1.5 in LG and 1.4 in HG (*p* = 0.50). QoL improved significantly comparing preoperative status to 1 year after operation in both groups since the bodily pain score increased by 7.8 points (*p* < 0.001) and physical functioning by 4.3 points (*p* = 0.014).

**Conclusion:**

Long-term follow-up is needed to demonstrate the potential advantage of a hybrid operation with fascial defect closure. Both techniques had low hernia recurrence rates 1 year after operation. LIVHR reduces chronic pain and physical impairment and improves QoL.

**Trial Registry**: Clinical trial number NCT02542085.

In incisional ventral hernia repair, the laparoscopic method (LIVHR) has gained popularity since it reduces postoperative morbidity and hospital stay compared to the open approach [1]. However, common problems with LIVHR include bulging, seroma formation and failure to restore of the abdominal wall function [1–4]. Seroma infection can lead to mesh removal [5] and hernia recurrence [6, 7]. Apart from recurrence, chronic pain and QoL are important outcome variables for ventral hernia repair. Chronic pain may be of significant concern in many patients, leading to prolonged consumption of analgesics and restriction in daily activities. The incidence of chronic pain after LIVHR ranges between 1.3 and 14.7% [8–10]. Even with various benefits, LIVHR has not overcome the open method regarding hernia recurrence [11–14] or chronic pain [15, 16]. Because of these issues, LIVHR combined with the defect closure (hybrid or intraperitoneal onlay mesh [IPOM-Plus]) has been introduced in several studies with promising outcome [7, 17, 18]. Hybrid method has been associated with a lower recurrence rate compared with standard LIVHR (0–4.7% [7, 16, 17] vs. 3.8–16.7% [9, 16, 19–21]). It seems that closing the hernia defect also lowers the risk for chronic pain [17].

One obvious goal in ventral hernia repair is to improve patients’ QoL. Only a few studies have measured the influence of LIVHR on QoL [22–24]. According to these outcomes, LIVHR appears to reduce chronic pain and physical impairment and improves long-term QoL.

This trial compares hybrid and standard LIVHR with primary outcome measures of seroma formation at 1 month and hernia recurrence at the 1-year follow-up. Our short-term results have been published previously [25]. The present study aims to find out whether a hybrid operation with diminished postoperative seroma formation has any impact on hernia recurrence compared to LIVHR. As a secondary hypothesis, we evaluated whether closing the defect lowers postoperative chronic pain and improves QoL.

## Patients and methods

The present study is the latter part of a prospective randomised controlled multicentre study comparing laparoscopic and hybrid technique in incisional ventral hernia repair, registered in Clinicaltrials.gov as NCT02542085. Eleven Finnish hospitals participated in the study. The perioperative parameters and short-term postoperative complications have been published previously [25]. The study was approved by the local ethical committee of each hospital.

From November 2012 to May 2015, following informed consent protocols, adult patients (18–80 years) undergoing incisional ventral hernia repair (IVHR) using a Parietex® Composite mesh (Covidien) were randomly assigned to receive either a conventional laparoscopic mesh repair or a hybrid repair. In the hybrid group the hernia sack was resected, and the fascial defect was closed with a slowly absorbing monofilament suture (0–0 Maxon® or PDS®) through a minilaparotomy incision before the standard laparoscopic intraperitoneal onlay mesh repair. Patient-related parameters were recorded in the outpatient clinic preoperatively. Peri- and postoperative details were collected, and follow-up visits were arranged after 1 month and 1 year. Postoperative complications were graded according to the Clavien–Dindo scale [26]. Specific characteristics of the surgical technique are described in our previous article [25].

A follow-up ultrasound examination was performed to show possible seromas or recurrent hernias. Ultrasound examination was performed in the supine position during rest and Valsalva’s manoeuvre [27]. The sizes of seromas and recurrent hernias were measured.

The registration of pain with VAS was performed during the postoperative hospital stay and in the follow-up controls.

The QoL was measured using the generic SF-36 short form questionnaire [28]. The SF-36 measures eight scale scores: physical functioning, role functioning (physical), role functioning (emotional), bodily pain, general health, vitality, social functioning, emotional wellbeing and changes in health. For all scales, higher scores represent better function/outcome. The SF-36 was completed by the patients preoperatively, after 1 month and after 1 year.

Patients’ satisfaction with the cosmetic outcome was measured using a scale from 1 to 10 (1 = totally unsatisfied and 10 = totally satisfied) after 1 year from surgery.

## Study outcome

The main outcome measure of this study is hernia recurrence 1 year after surgery. The secondary measures are long-term complications, pain (VAS) and QoL. The randomisation process and its implementation are presented elsewhere [25].

## Statistical analysis

The current study compares the results of hybrid and laparoscopic techniques on hernia recurrences at the 1-year follow-up. The primary endpoint is hernia recurrence at 1 year postoperatively. According to sample size calculation, assuming 6% difference (2% in HG vs. 8% in LG, α = 0.05, power = 0.80, and a drop-out rate of ~ 20%) in the hernia recurrence rate at the 1-year follow-up, 200 patients per group needed to be randomised.

Summary measurements are presented as mean with standard deviation unless otherwise stated. Between-group comparisons were performed by *χ*^2^ test or Fisher’s exact test (categorical variables) and by Student’s *t* test or Mann–Whitney *U* test (continuous variables). All analyses were done according to the intention to treat (ITT) principle unless otherwise stated. Two-tailed *p* values < 0.05 were considered statistically significant. Repeatedly measured continuous variables were analysed using a linear mixed model (LMM), in which patients were selected randomly and the covariance pattern was chosen according to Akaike’s information criteria. Effect sizes with 95% confidence intervals (CI) for a 1-year outcome are presented according to LMM. Analyses were performed by SPSS for Windows (IBM Corp. Released 2013. IBM SPSS Statistics for Windows, Version 22.0. Armonk, NY: IBM Corp.) and SAS (version 9.4, SAS Institute Inc., Cary, NC, USA).

## Results

**Fig. 1:**
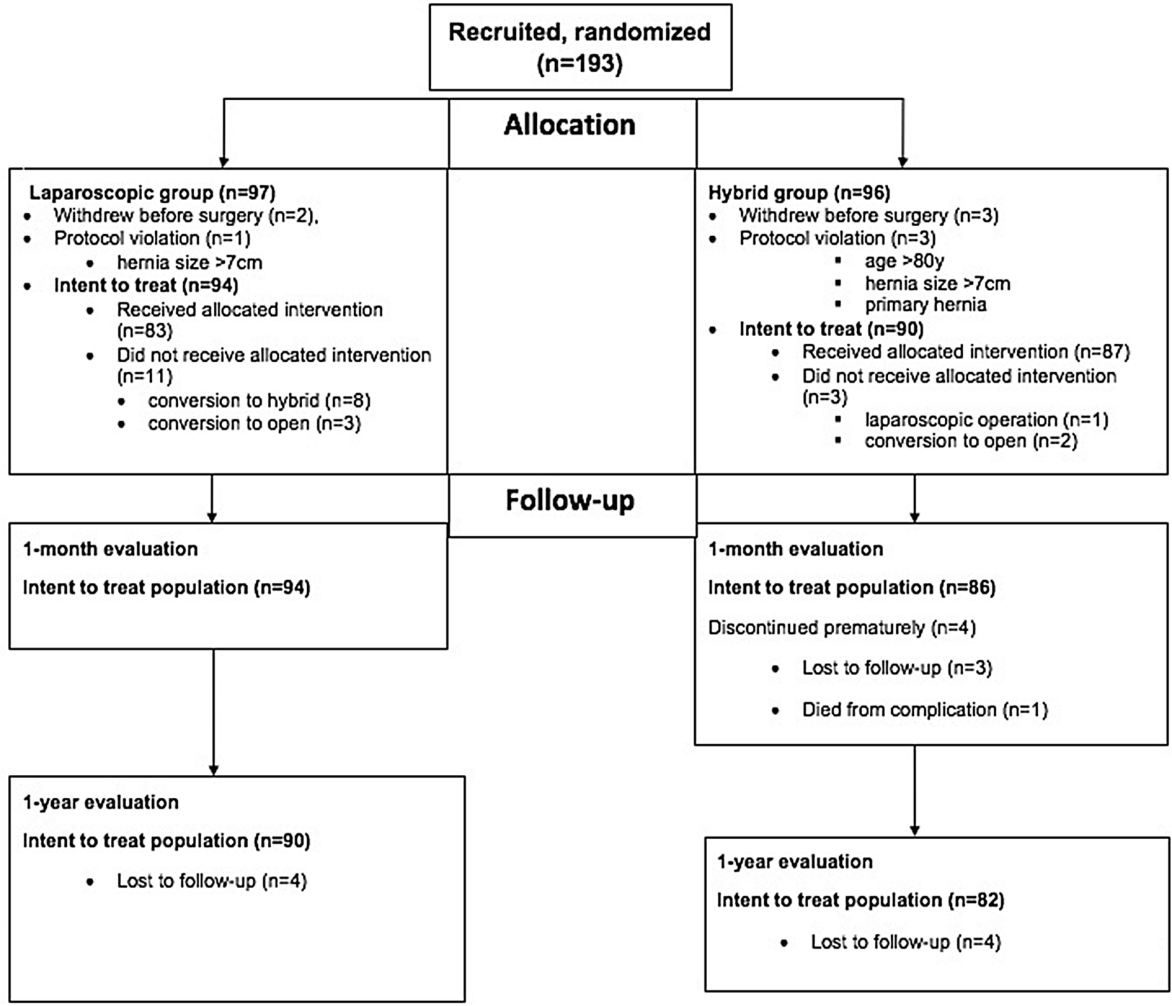
Flow diagram

Altogether, 193 patients with incisional ventral hernia were randomly assigned to either the laparoscopic group (LG) or the hybrid group (HG). From these 193 patients, 94 patients in LG and 90 in HG underwent surgery and were analysed (Table 1). Further, 90 patients from LG and 82 from HG returned for the 1-year-control visit. The flow chart is shown in Fig. 1.

**Table 1:** Baseline characteristics

	Laparoscopic group *n* = 94	Hybrid group *n* = 90
Age [years, mean (SD)]	57 (SD 11.4)	60 (SD 12.8)
Gender [*n* (%)]		
Female	56 (59.6)	55 (61.1)
Male	38 (40.4)	35 (38.9)
BMI [kg/m^2^ (SD)]	30.2 (SD 4.4)	29.2 (SD 4.2)
ASA class [*n* (%)]		
1	10 (10.9)	12 (13.6)
2	48 (52.2)	44 (50)
3	34 (37.0)	32 (36.4)
Smoking [*n* (%)]	17 (18.1)	11 (12.2)
Preoperative pain	74 (78.7)	69 (76.7)
Radiological findings		
Hernia defect size [cm^2^, mean (SD)]	13.2 (SD 11.1)	10.5 (SD 8.9)
Number of hernias [*n* (%)]		
1	66 (70.2)	65 (73)
2	17 (18.1)	16 (18.0)
≥ 3	11 (11.7)	8 (9)

Nominal variables are reported as counts and percentages (in parentheses); continuous variables are reported as mean and standard deviation

*ASA* American Society of Anesthesiologists

The perioperative parameters and short-term postoperative complications up to the 1-month-control visit have been reported in our previous publication [25].

At the 1-year-control visit, we found no difference in hernia recurrence between the study groups. Altogether, 11 recurrent hernias were found radiologically, resulting in a recurrence rate of 6.4% (11/172); 6 (6.7%) in LG and 5 (6.1%) in HG (difference 0.6%, 95% CI – 7.6 to 8.6%, *p* > 0.9). Re-hernioplasty is planned for only two patients; the others are asymptomatic and continue with conservative care. No specific predicting or predisposing factors were found related to recurrence in either patient-related factors or peri- or postoperative data.

Clinical outcome at the 1-year point is shown in Table 2. Patients in HG were more asymptomatic (76/82, 93%) and pain-free (4/82, 5%) compared to LG (74/90, 82%) and (12/90, 13%), but without a statistically significant difference. Patients were satisfied with the cosmetic results of the operations: on a scale from 1 to 10, the mean value in both groups was 9.

**Table 2:** One-year clinical outcome in the study groups

	Laparoscopic group *n* = 90	Hybrid group *n* = 82	Difference^a^	95% CI for the difference	*p* Value
Symptoms [*n* (%)]					
None	74 (82)	76 (93)	– 10.5	– 20.4 to – 0.3	0.066
Bulging	6 (7)	4 (5)	1.8	– 6.1 to 9.5	0.7
Pain	12 (13)	4 (5)	8.5	– 0.5 to 17.5	0.07
VAS [mean (SD)]	1.5 (1.2)	1.4 (1.1)	0.1	– 0.2 to 0.5	0.5
Clinical findings [*n* (%)]					
Palpable mass	22 (24)	12 (16)	8.6	– 3.6 to 20.3	0.19
Recurrent hernia	4 (5)	3 (4)	0.8	– 6.3 to 7.7	> 0.99
Radiological findings					
Hematoma/seroma	12 (13)	5 (6)	7.2	– 2.0 to 16.5	0.13
Hernia sack remnant	7 (8)	3 (4)	4.1	– 3.5 to 11.9	0.34
Recurrent hernia confirmed	6 (7)	5 (6)	0.6	– 7.6 to 8.5	> 0.99
Re-hernioplasty	1 (1)	1 (1)	0.1	– 5.6 to 4.9	> 0.99
Cosmetic evaluation [mean (SD)]	8.8 (1.5)	8.9 (1.6)	– 0.08	– 0.55 to 0.39	0.74

Nominal variables are reported as counts and percentages (in parentheses); continuous variables are reported as the mean and standard deviation

*VAS* visual analogue scale; pain severity was estimated by the VAS scale from 1 to 10

^a^Difference in percentage units

**Fig. 2:**
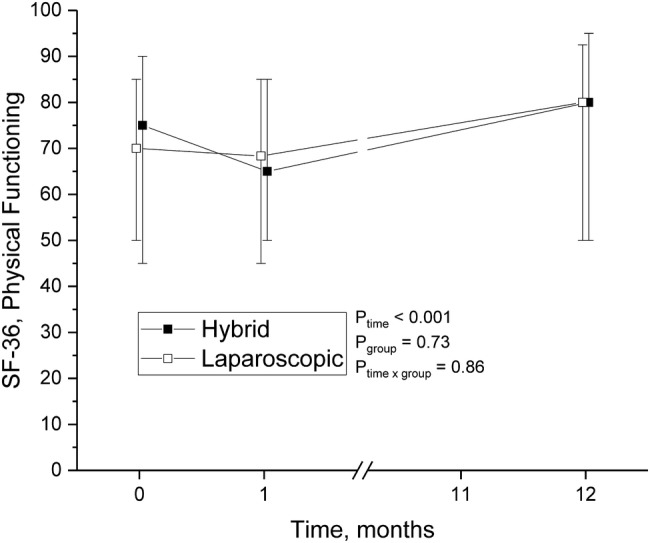
Physical functioning

**Fig. 3:**
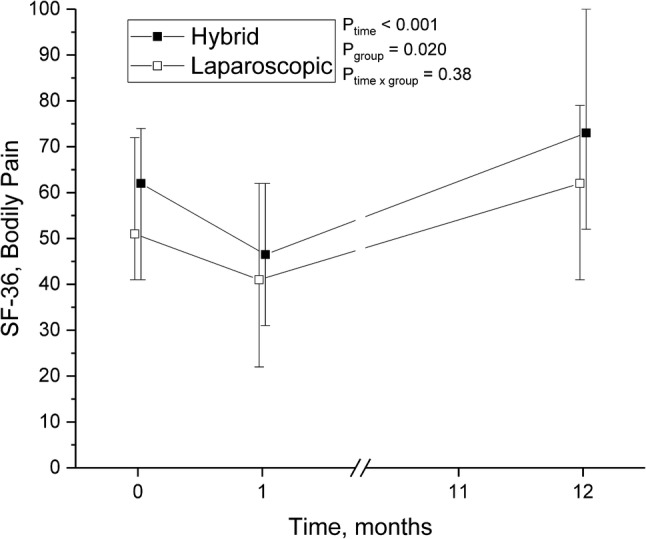
Bodily pain

The QoL questionnaires were gathered at three points: preoperatively, at 1-month and 1-year visits. The main finding was that operative treatment of hernia, whether laparoscopic or hybrid, significantly improved patients’ QoL when comparing preoperative time to 1 year after operation (Table 3). In particular, scores in physical functioning increased by 4.3 points (*p* = 0.014) (Fig. 2), and bodily pain by 7.8 points (*p* < 0.001) (Fig. 3) during the follow-up. By coincidence, compared to LG, the patients in HG reported markedly less pain before the hernia repair, and this difference remained after 1 year. Otherwise, no differences between the operative groups were seen. The pain scores 1 year after operation were very low in both groups; the mean VAS was 1.5 in LG and 1.4 in HG (*p* = 0.5).

**Table 3:** Pre- and postoperative SF-36 scores comparing results in laparoscopic and hybrid group

SF-36 items	Preoperatively	1-Month postoperatively	1-Year postoperatively	Difference (95% CI)^a^	*p* Time	*p* Group	*p* Time* group
Mental health (MH)							
LG	74.7 (SD 17.7)	71.4 (SD 21.2)	77.9 SD (16.8)	3.0 (– 3.0 to 9.0)	0.056	> 0.90	0.18
HG	76.0 (SD 20.3)	74.4 (SD 19.7)	75.2 SD (20.56)				
Role physical (RP)							
LG	48.9 (SD 40.9)	30.2 (SD 38.0)	61.7 (SD 42.4)		< 0.001	0.43	0.10
HG	60.7 (SD 41.0)	29.8 (SD 37.3)	61.4 (SD 44.0)	0.1 (– 12.7 to 12.9)			
Bodily pain (BP)							
LG	54.0 (SD 24.8)	44.6 (SD 26.6)	59.1 (SD 25.4)	– 10.5 (– 18.4 to – 2.7)	< 0.001	0.02	0.38
HG	59.2 (SD 24.3)	49.9 (SD 22.8)	69.8 (SD 25.5)				
General health (GH)							
LG	59.4 (SD 18.2)	59.6 (SD 19.8)	58.2 (SD 18.9)	– 0.4 (– 6.8 to 5.9)	0.31	0.76	0.47
HG	57.7 (SD 24.0)	59.1 (SD 20.7)	57.9 (SD 21.8)				
Vitality (VT)							
LG	62.4 (SD 20.2)	57.5 (SD 24.3)	63.8 (SD 22.7)	– 0.7 (– 7.7 to 6.3)	0.073	0.59	0.53
HG	62.8 (SD 22.6)	62.0 (SD 22.8)	63.5 (SD 23.2)				
Social functioning (SF)							
LG	75.3 (SD 25.0)	68.0 (SD 27.7)	81.9 (SD 23.1)	3.2 (– 4.9 to 11.4)	0.004	0.50	0.08
HG	78.2 (SD 27.3)	76.5 (SD 23.5)	78.3 (SD 29.8)				
Role emotional (RE)							
LG	61.4 (SD 43.1)	59.7 (SD 42.3)	71.14 (SD 37.3)	2.9 (– 9.9 to 15.7)	0.010	0.85	0.36
HG	66.0 (SD 40.5)	56.7 (SD 42.4)	66.7 (SD 40.2)				
Physical functioning (PF)							
LG	67.3 (SD 23.6)	64.6 (SD 26.5)	71.4 (SD 26.4)	– 2.2 (– 10.0 to 5.6)	< 0.001	0.73	0.86
HG	67.5 (SD 25.8)	65.4 (SD 23.4)	71.9 (SD 26.2)				

*Data from 74 patients in LG and 69 patients in HG

^a^Mean difference between study groups with 95% CI at 1 year

## Discussion

This study is the first randomised trial comparing laparoscopic and hybrid incisional hernia operations. The research was performed with the cooperation of 11 hospitals in Finland.

The main purpose was to explore whether, by using the hybrid technique in incisional hernia repair, the postoperative seroma formation could be diminished, which may lead to a decrease in the hernia recurrence rate. At 1 month after surgery, the patients who received the hybrid technique suffered significantly less from seroma formation than patients who received laparoscopy (39 [45.3%] vs. 63 [67%], *p* = 0.004), supporting our hypothesis. However, this outcome had no impact on hernia recurrence at 1 year after operation. When comparing patients who had seroma at 1-month control and were diagnosed with a recurrent hernia 1 year after the operation, the difference was only 1.9% in favour of the hybrid group. No correlation between hernia recurrence and seroma formation or other known risk factors (BMI > 30, smoking, postoperative complications) was seen, presumably due to a small number of recurrent hernias.

According to recent studies, fascial closure combined with IPOM repair leads to recurrence rates as low as 0.0–4.8% [7, 16, 17]. In only one of these studies [17], the hernia recurrence was evaluated routinely by computed tomographic scans at each 3-month visit. Others were retrospective or descriptive analyses. A common problem, bulging related to IPOM repair, can manifest as a pseudo-recurrence and therefore mimic a recurrent hernia without radiological confirmation [3]. In our study, seven recurrences out of 172 (4%) patients were found in clinical evaluation. Altogether, 11 (6.4%) recurrences were confirmed by radiological examination, 6 (7%) in the laparoscopic group and 5 (6%) in the hybrid group. Only two of our patients with recurrent hernia had symptoms, such as pain and bulging, and were therefore referred for re-hernioplasty. Under these circumstances, clinically relevant hernia recurrence rates were as follows: laparoscopic group 1.1%, hybrid group 1.2%, and 1.2% amongst all study patients.

Guidelines for the laparoscopic treatment of ventral and incisional abdominal wall hernias, published by the International Endohernia Society (IEHS) [4], introduced a recommendation for tension-free fascial closure to be combined with standard laparoscopic hernia repair (augmentation repair). As stated in the guidelines and shown in our study, the hybrid method eliminates bulging and decreases the seroma size and incidence, hence keeping the potential infection risk low. Against our hypothesis, we did not find a difference in hernia recurrence rates between the study groups, despite the difference in seroma incidence. This result is, however, in line with a recent review of studies comparing standard IPOM and hybrid repair [29]. According to the literature, most of the hernia recurrences occur during the first 2 years after the repair [1, 30, 31]. In this aspect, a 1-year follow-up is rather short, and therefore the follow-up for the patients included in this study will continue up to 5 years.

In line with other studies [22, 23], our study shows that hernia repair improves patients’ quality of life in the long run, assuming that the beneficial effect found at 1 year will last. A mild deterioration related to the recovery phase was seen at 1 month after the operation.

One drawback of our study is that we failed to reach the estimated sample size of 400 patients, even though we extended the recruitment period from 1 year to 30 months. As augmentation repair increased in popularity during the study time and was recommended by the recent IEHS guidelines, surgeons became less enthusiastic in recruiting patients into the study. Furthermore, we did not have a complete screening log from all the centres involved in the study, which may lead to selection bias.

## Conclusion

Both techniques—standard laparoscopic hernia repair and the hybrid method—have a low hernia recurrence rate at 1 year after the operation. Long-term follow-up is needed to demonstrate the possible advantage of the hybrid operation with fascial defect closure. Laparoscopic hernia repair reduces chronic pain and physical impairment and improves quality of life significantly.
